# Integration of Biological Networks for *Acidithiobacillus thiooxidans* Describes a Modular Gene Regulatory Organization of Bioleaching Pathways

**DOI:** 10.3389/fmolb.2019.00155

**Published:** 2020-01-10

**Authors:** María Paz Cortés, Vicente Acuña, Dante Travisany, Anne Siegel, Alejandro Maass, Mauricio Latorre

**Affiliations:** ^1^Center for Mathematical Modeling, Universidad de Chile and UMI CNRS 2807, Santiago, Chile; ^2^Center for Genome Regulation, Universidad de Chile, Santiago, Chile; ^3^IRISA, UMR 6074, CNRS, Rennes, France; ^4^INRIA, Dyliss Team, Centre Rennes-Bretagne-Atlantique, Rennes, France; ^5^Department of Mathematical Engineering, Universidad de Chile, Santiago, Chile; ^6^Laboratorio de Bioinformática y Expresión Génica, INTA, Universidad de Chile, Santiago, Chile; ^7^Instituto de Ciencias de la Ingeniería, Universidad de O'Higgins, Rancagua, Chile

**Keywords:** *Acidithiobacillus thiooxidans*, biological networks, co-regulation, bioleaching, biotechnology

## Abstract

*Acidithiobacillus thiooxidans* is one of the most studied biomining species, highlighting its ability to oxidize reduced inorganic sulfur compounds, coupled with its elevated capacity to live under an elevated concentration of heavy metals. In this work, using an *in silico* semi-automatic genome scale approach, two biological networks for *A. thiooxidans* Licanantay were generated: (i) An affinity transcriptional regulatory network composed of 42 regulatory family genes and 1,501 operons (57% genome coverage) linked through 2,646 putative DNA binding sites (arcs), (ii) A metabolic network reconstruction made of 523 genes and 1,203 reactions (22 pathways related to biomining processes). Through the identification of confident connections between both networks (V-shapes), it was possible to identify a sub-network of transcriptional factor (34 regulators) regulating genes (61 operons) encoding for proteins involved in biomining-related pathways. Network analysis suggested that transcriptional regulation of biomining genes is organized into different modules. The topological parameters showed a high hierarchical organization by levels inside this network (14 layers), highlighting transcription factors CysB, LysR, and IHF as complex modules with high degree and number of controlled pathways. In addition, it was possible to identify transcription factor modules named primary regulators (not controlled by other regulators in the sub-network). Inside this group, CysB was the main module involved in gene regulation of several bioleaching processes. In particular, metabolic processes related to energy metabolism (such as sulfur metabolism) showed a complex integrated regulation, where different primary regulators controlled several genes. In contrast, pathways involved in iron homeostasis and oxidative stress damage are mainly regulated by unique primary regulators, conferring Licanantay an efficient, and specific metal resistance response. This work shows new evidence in terms of transcriptional regulation at a systems level and broadens the study of bioleaching in *A. thiooxidans* species.

## Introduction

*Acidithiobacillus thiooxidans* belongs to the *Acidithiobacillia* class of proteobacteria (Williams and Kelly, 2013). It is an autotrophic Gram-negative bacterium that obtains energy from the oxidation of reduced inorganic sulfur compounds (RISC). *Acidithiobacillus thiooxidans* capacity to produce sulfuric acid, especially during the control of biochemical steps related to elemental sulfur oxidation pathways and the acidification of the media (Mohapatra et al., 2008) have positioned this bacterium as one of the most studied organism in the field of bioleaching processes (Chen et al., 2015; Yan et al., 2015; Quatrini et al., 2017; Zhou et al., 2017).

Recently, *A. thiooxidans* Licanantay was presented as one of the most relevant participants of a consortium of five natural copper-bioleaching acidophilic bacteria (Latorre et al., 2016). This bacterium was isolated directly from a copper mine in the north of Chile. Its genome sequence revealed an elevated number of genes associated with RISC oxidation: several HDR complex genes, two gene copies for the sulfur oxidizing complex (Sox) and one archaeal type sulfur oxygenase reductase gene (*sor*) (Travisany et al., 2014), attributes directly correlated with its efficiency in copper recovery. In addition, Licanantay has an elevated capacity to survive under elevated concentrations of copper, arsenic, and chloride in relation to other biomining species and produces high quantities of glutathione (Martínez et al., 2013), a crucial metabolite directly or indirectly related to iron and RISC oxidation in bioleaching species.

A complete genome comparative analysis between nine draft genomes of *A. thiooxidans* postulates that the genetic diversity of this species might be correlated with geographic location and geochemical conditions (Zhang et al., 2016). In this study, the comparison between Licanantay and the reference strain AT19377 reaffirms the fact that the Chilean bacterium has a higher number of unique genes, which may confer an adaptive advantage to extreme environmental conditions for Licanantay compared to other *A. thiooxidans* strains.

In addition, a set of environmental resistance elements and metabolic pathways presumed relevant to its performance in bioleaching processes have been assigned to this bacterium, most of them related to the oxidation of RISC, metal resistance, biofilm formation, and energy production (Latorre et al., 2016). These results position *A. thiooxidans* Licanantay as an excellent model to study genomic and metabolic features in terms of gene regulation and metabolic pathways related to the adaptation of this bacterium to the environment of a copper mine.

Using bioinformatics tools in combination with a manual curation of regulatory patterns, a great amount of information can be extracted from the genome sequence and further summarized in an affinity transcriptional regulatory network (Balleza et al., 2008). These models depict the total set of statistically significant affinity relations between annotated transcription factors and their binding sites in promoter regions of operons. It is important to remark that this affinity relation does not necessarily imply that the regulatory relation is effectively used for a given set of conditions. Indeed, the regulatory process also depends on other factors that vary depending on the conditions imposed on the cell, and only expression experiments can confirm such relation (Potash, 2007). However, the strategy of generating affinity networks has been widely used in bacterial organisms as a starting point to identify a global regulatory organization. Affinity networks provide relevant information about the topological configuration of gene regulation at a system level and allows the importance of specific regulatory elements and its putative gene/operons targets to be identified (Balázsi et al., 2008; Latorre et al., 2014; Yus et al., 2019).

On the other hand, the study of a metabolic network is key to gaining insight regarding phenotypic features of an organism. The reconstruction of metabolic networks at the genome scale, i.e., incorporating all available information, allows us to have a global, and comprehensive picture of metabolism. These genome-scale reconstructions are considered specific knowledge repositories of studied organisms where information regarding their metabolism is organized and new data can be later integrated (Feist et al., 2009). This can be particularly useful to guide and contribute to the systematic study of less-studied organisms, as is the case of biomining organisms in general.

In this work, using a systems biology approach, genome-scale metabolic, and regulatory networks were integrated. The main objective of this article was to generate information on the transcriptional mechanism able to control the expression of elements involved in metabolic pathways related to bioleaching in *A. thiooxidans* Licanantay. To this end, the minimal configuration able to maintain bacterial-relevant functions was described and we showed that this gene regulatory organization strongly depended on different types of modules.

## Results and Discussion

### *A. thiooxidans* Licanantay Affinity Transcriptional Regulatory Network

**Figure 1:**
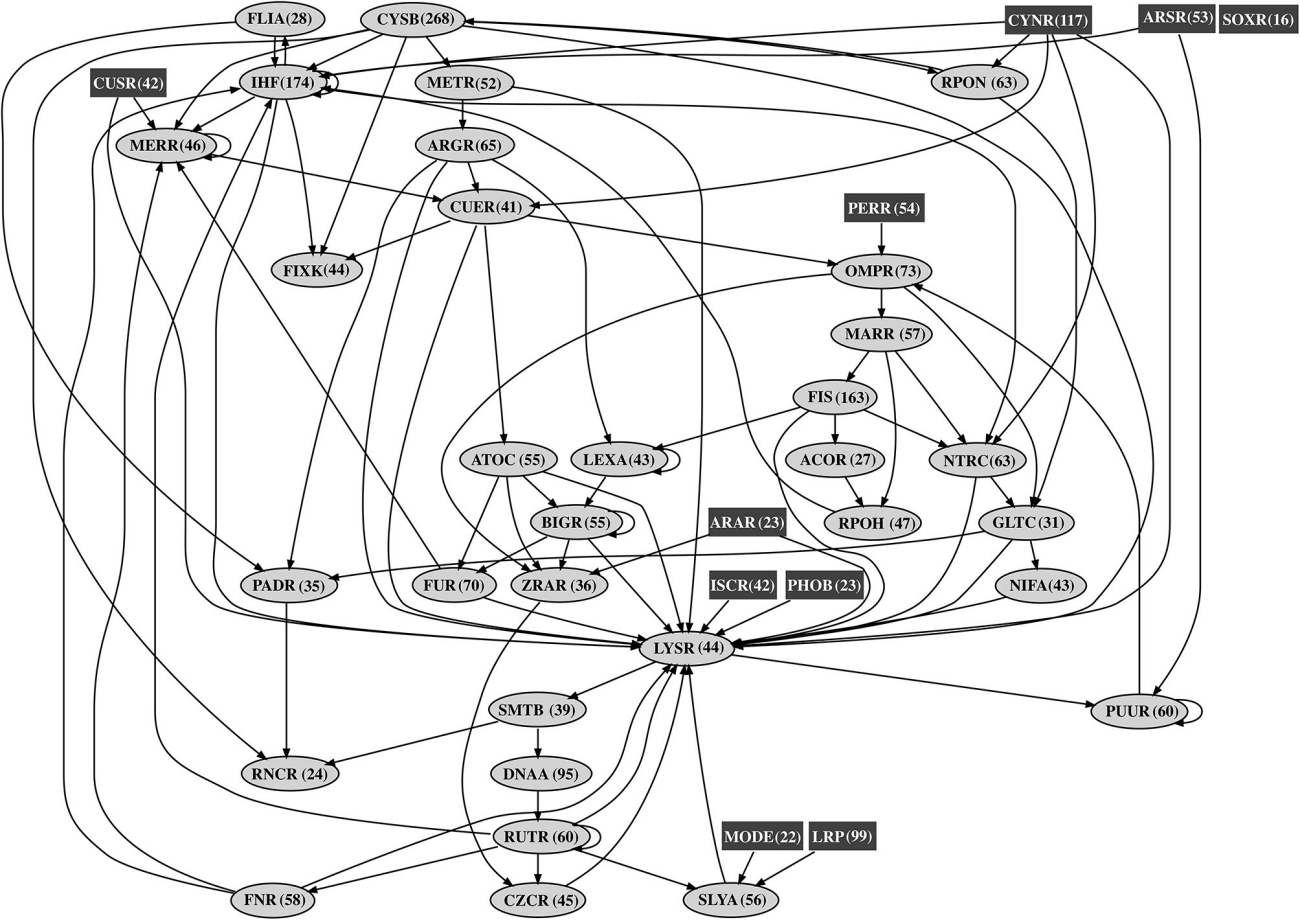
*Acidithiobacillus thiooxidans* Licanantay affinity transcriptional regulatory network. The figure shows the interconnectivity (black arrows) between transcriptional factor nodes. Rectangular nodes (dark gray) correspond to transcriptional factors not regulated by others (origons). Oval nodes (light gray) represent transcriptional factors member of chain regulatory cascades. The number in parenthesis next to each transcriptional factor name is the number of operon targets for that transcriptional factor in the affinity network.

In order to understand the global transcriptional regulatory organization in *A. thiooxidans* Licanantay, a genome-scale affinity transcriptional regulatory network was generated. The complete model had a genome coverage of 57% and was composed of 1,543 nodes (42 corresponding to transcriptional factor nodes) and 2,646 arcs (putative binding sites) (Figure 1). The degree distributions (in-degree, out-degree, and total degree) showed a typical shape in which most nodes have a low degree and only a few nodes are highly connected (Albert, 2005). This characteristic is typical in power low distributions observed in other bacterial transcriptional regulatory networks. In terms of the interconnectivity between transcriptional factors, the network model contains at least three types of regulators (Schröder and Tauch, 2010). First, a global set of regulators, like LysR (global metabolism), and IHF (DNA structural organization), which are highly interconnected in the network. As shown in Figure 1, these two regulators present a multi-level regulation cascade structure, representative of a classical chain transcriptional regulatory process. Second, a set of master regulators (moderately connected), such as AtoC (acetoacetate metabolism) and MerR (metal resistance). Acetoacetate was identified as a biofilm inhibitor (Horne et al., 2018), an important bacterial process during ore bioleaching (Bellenberg et al., 2014). The MerR family is highly conserved in other biomining organisms, including strains of *At. ferrooxidans* (Hödar et al., 2012). Finally, the third class corresponded to local regulators, highlighting the proteins Fur and CueR (also a MerR family member), controllers of metal homeostasis and oxidative stress damage, two main cellular processes considering the mining environment where Licanantay was isolated (Latorre et al., 2016), during oxidative dissolution, autotrophic organisms are able to use ferrous iron and reduced sulfur compounds as electron donors. In addition, the model contained a total of 10 regulators not controlled by other transcription factors (isolated). This type of element is called Origons (Balázsi et al., 2005) and represents topological units of environmental signal processing, able to directly transduce stimulus into gene expression control (direct and fast response). Inside this group, Licanantay had the CusR transcription factor, one of the main regulators of copper homeostasis in Gram-negative bacteria (Rensing and Franke, 2007). Considering the elevated concentration of copper in the mine, the presence of the CusR origon gives Licanantay an efficient and fast control over copper homeostasis, in particular over the expression of CopA ATPase involved in this metal efflux (Solioz and Stoyanov, 2003). Finally, the node with the highest out-degree (275) was CysB, making it one of the main Hubs inside the network. This regulator is known as a master regulator of genes encoding for proteins involved in sulfur metabolism, particularly, its assimilation (van der Ploeg et al., 2001) and also iron starvation (Imperi et al., 2010). For *A. thiooxidans* species, sulfur metabolism plays a crucial role in the acquisition of electrons for their autotrophic growth (Wang et al., 2018).

### *A. thiooxidans* Licanantay Metabolic Network

Efforts have been made to reconstruct the metabolic networks for a few bioleaching bacteria (Hold et al., 2009; Merino et al., 2010; Bobadilla Fazzini et al., 2013; Merino Santis et al., 2015). These reconstructions were developed with the objective of generating metabolic models that allow the prediction of growth rates in different scenarios through metabolic flux analysis. With the exception of *At. ferrooxidans* ATCC23270, for which a genome-scale reconstruction was built (Campodonico et al., 2016), bioleaching bacterial metabolic reconstructions corresponded to reduced and simplified representations of their networks in all cases. This was also the case for *A. thiooxidans* Licanantay, for which we previously built a small stoichiometric model used to predict its growth rate in different media containing different reduced sulfur compounds for oxidation (Bobadilla Fazzini et al., 2013). This model incorporates a total of 181 metabolic reactions associated with RISC oxidation, central metabolism, amino acids, and nucleotides biosynthesis pathways.

For the work presented here, we revisited the analysis of *A. thiooxidans* Licanantay metabolic network, this time aiming at a global genome-scale reconstruction in order to later link metabolic genes through the regulatory network of the bacteria. To do this, we followed a semi-automatic approach starting by a full genome re-annotation in order to make the most of the available data. This new annotation resulted in 564 unique Enzyme Commission (EC) numbers, 20% of which were absent from our previous annotation and an improved annotation of 81 genes previously identified as hypothetical protein coding genes.

Our current genome-scale metabolic network reconstruction was made of 1,203 reactions, associated with 523 genes coding for enzymes and transport proteins. This reconstruction included all enzymatic reactions incorporated in the previous stoichiometric model as well as additional relevant reactions and pathways, e.g., the biosynthesis of spermidine, a metabolite that has been linked to sulfur-oxidation in a previous metabolomic study on this bacterium (Martínez et al., 2013) as well as to pH homeostasis and oxidative stress management (Samartzidou et al., 2003; Ferrer et al., 2016).

Interestingly, sulfur metabolism and siderophore biosynthesis were both highly connected in the metabolic network. Sulfur metabolism is directly connected to the capacity to produce cysteine in bacterial species. This amino acid can be used to synthetize Fe-S clusters, the principal co-factor of the HDR complex. Competition for iron occurs in acidic environments, where the capacity to produce and recognized different siderophores could be an adaption to respond to different iron concentrations (Bonnefoy and Holmes, 2012). In addition, biofilm processes in the model are related to routes involved in lysine degradation. This amino acid inhibits coaggregation and synergy in biofilm formation (Sharma et al., 2005; Okuda et al., 2012). The capacity of biomining organisms to produce biofilms is one of the critical and most studied areas of bioleaching. The active presence of lysine degradation pathways in Licanantay, supports the high capacity of this bacterium to recover copper during the process.

In previous work, a number of metabolic processes were linked to the bioleaching capacity of a bacterial consortium that has *A. thiooxidans* Licanantay as one of its members (Latorre et al., 2016). These processes included known key bioleaching steps, such as iron and RISC oxidation as well as related metabolic features such as sulfur assimilation, biosynthesis of essential components and precursors, electron transfer and energy generation, and biofilm formation.

**Figure 2:**
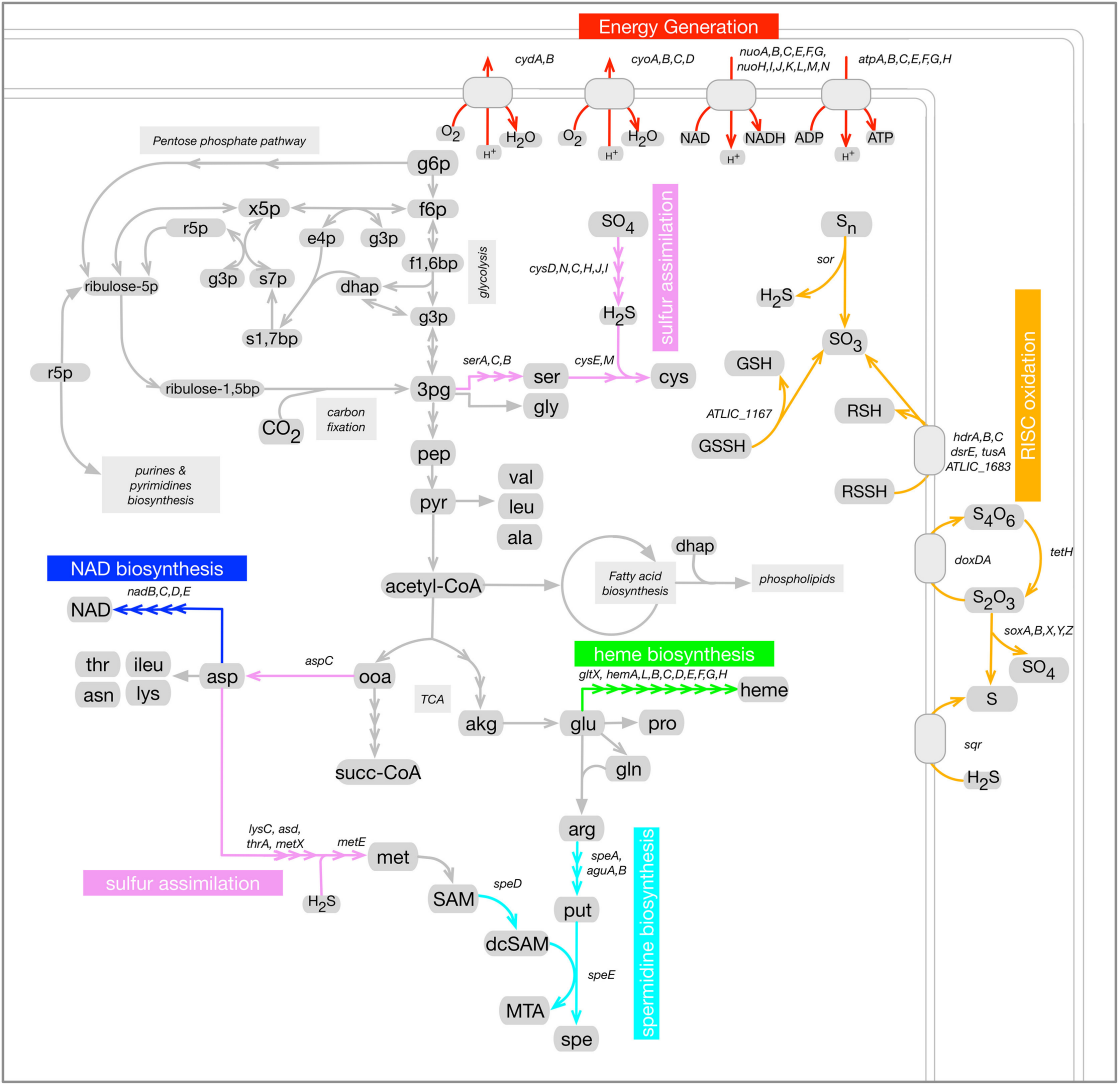
Selected metabolic pathways related to bioleaching in the context of *A. thiooxidans* Licanantay metabolic network (sub-categories). Six metabolic processes were selected for this study: RISC oxidation (orange); Sulfur assimilation (violet); Energy generation (red); heme biosynthesis (green); spermidine biosynthesis (cyan); and NAD biosynthesis (blue). Genes associated to each of these pathways are listed next to the corresponding reactions.

The next step in the current study, was to consider a subset of these metabolic categories to focus our analysis of *A. thiooxidans* regulation on particularly relevant processes related to bioleaching that could be subject to co-regulation. This subset was composed of six sub-categories, selected because they corresponded to well-described bioleaching metabolic pathways part of the *A. thiooxidans* metabolic network. Figure 2 shows these pathways in the context of the *A. thiooxidans* global metabolic network. They corresponded to RISC oxidation, sulfur assimilation, heme, NAD, and spermidine biosynthesis processes.

RISC oxidation (orange pathways in Figure 2) by sulfur-oxidizing bacteria such as *A. thiooxidans* is key for bioleaching operations. It results in the release of sulfuric acid which helps maintain the acidic condition required for bioleaching to occur. RISC is the only electron donors utilized by *A. thiooxidans*. Thus, sulfur oxidation was strongly linked to general metabolic pathways related to energy generation (depicted in red in Figure 2) that involve steps to harness energy through proton gradient and reducing power generation. Given that NAD(H) is a main reducing power carrier, its biosynthesis pathway was also considered in this analysis (blue pathway in Figure 2).

For sulfur oxidizers, as has been previously pointed out for *At. ferrooxidans* (Valdés et al., 2003), a balance should take place in the use of sulfur as an energy source and in the assimilation processes. Moreover, RISC oxidation is a complex process whose associated metabolic pathways have not being fully elucidated to date. Different pathways have been proposed for the *Acidithiobacillus* species (Wang et al., 2018) including a pathway that involves the assimilation enzymes APS kinase and PAPS reductase (Yin et al., 2014). Based on these considerations sulfur assimilation metabolic pathways were also considered in this analysis (pink pathways in Figure 2). Spermidine biosynthesis depicted in light blue in Figure 2, was also included given the previously mentioned link of this metabolite to sulfur oxidation.

Finally, the biosynthesis of heme was also included in this pathway selection (green pathways in Figure 2). Heme is an essential component of several proteins involved in electron transport chains which are key for *A. thiooxidans* energy generation. Heme is also a cofactor of enzymes involved in oxidative damage protection (Frankenberg et al., 2003). Minerals, which are abundant in bioleaching environments, are known to promote the formation of ROS species (Schoonen et al., 2006; Cárdenas et al., 2012), making protection mechanisms against them essential for *A. thiooxidans* survival. Additionally, as an iron-containing cofactor heme plays a role in iron homeostasis.

### Co-regulatory Integrative Network Analysis

As stated above, the affinity transcriptional network represents the set of all transcriptional regulatory relations between all transcription factors annotated in the genome and their putative target operons. Each relation was represented in the network by a directed arc from the operon coding for the regulator to the target operon (which can also code for another regulator). Thus, indirect regulation of the bioleaching sub-category via regulatory cascades can be defined as paths in the network.

It is important to declare that the set of arcs in the affinity transcriptional network is considered as an overrepresentation of the true transcriptional regulations occurring in the bacterium (Acuña et al., 2016). There are two main reasons for this overrepresentation. The first is purely methodological: some of the relations are simply false positives of the method that identifies binding sites. The second one is biological: even in the case that the transcription factors could effectively bind in a promoter region of a specific operon, bacteria only activate or repress this regulatory mechanism as required according to environmental conditions.

Considering these two statements, in order to give a new layer of likelihood to the regulatory relations effectively occurring in Licanantay for a given set of conditions, a method that selects feasible paths (i.e., regulatory cascades) was applied (Acuña et al., 2016). Under a parsimony principle, the method considers an arc between a transcription factor and its operon target as confident when it is part of a topological substructure (called V-shape) which is useful to coordinate the co-expression of operons in the same pathway.

**Figure 3:**
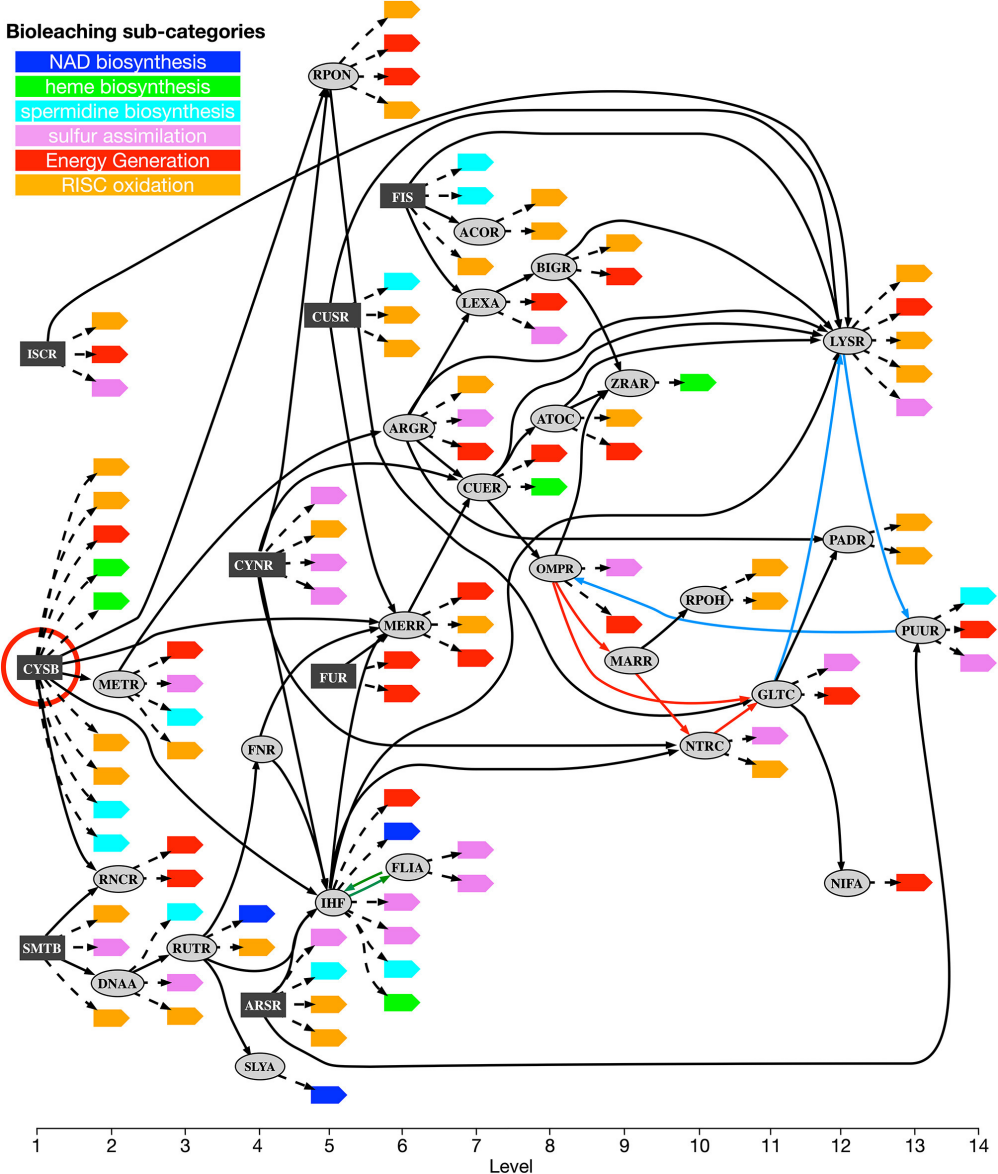
*Acidithiobacillus thiooxidans* Licanantay bioleaching co-regulatory network. Transcriptional factors in the co-regulatory network are depicted as rectangular (dark gray) and oval nodes (light gray). Rectangular nodes correspond to primary regulators while oval nodes are transcriptional factors member of chain regulatory cascades. Leaf nodes are target operons colored according to their metabolic bioleaching sub-categories. Solid arcs represent regulation between transcriptional factors and dotted arcs represent regulation of metabolic operons. Hierarchical levels are listed at the bottom of the figure. Red circle highlights CysB transcription factor. Colored arcs (red, green, and light blue) correspond to connections forming directed cycles in the network. There are three directed cycles: a small one between FLIA and IHF (green arcs) and two larger ones that share the path made by light-blue arcs. Thus, removing any green and light blue pair of arcs breaks all directed cycles (minimum feedback arc sets).

Using this method, sets of confident regulatory relations to coordinate the co-expression of operons in each one of the six selected metabolic sub-categories related to bioleaching processes were identified (see Materials and Methods for details). The union of these subnetworks can be considered a (transcriptional) “co-regulatory network” for bioleaching processes. This co-regulatory network is presented in Figure 3. The network was composed of 95 nodes, 34 of which were transcription factor families and 61 were metabolic operons from the bioleaching sub-categories, and 148 arcs, in which 57 corresponded to regulations between transcription factors and 91 to regulations of metabolic operons.

A first topological analysis of this network showed that it was almost hierarchical, presenting only a very small number of cycles (three in total), a classical organization for biological networks, which confer a directional regulatory collaboration between the transcription factors to the system. In fact, removing only two arcs broke every directed cycle in the network (i.e., the minimum feedback arc set had a size of two). This fact showed that the network can be organized into 14 levels with only two feedback arcs, as showed in Figure 3. The order of levels depended on which arcs were considered feedback arcs, and the figure represents only one possible configuration of these levels.

Even if the co-regulatory network was hierarchical (i.e., with almost no directed cycles inside), its organization was far from being like a tree graph. Indeed, if we consider only the 57 arcs between transcription factors, the number of them that need to be removed to obtain a tree was 24 or 42.1%. This means that the metabolic consequences of the regulation were not segregated by network level, having many arcs that cross from one branch to another or that jump directly to a distant level. This observation is also consistent with the fact that the regulation of metabolic operons of each sub-category was not separated by the hierarchy and, on the contrary, were spread along the entire network. This implies that the regulation process cannot be viewed as a sum of independent parts controlled by a central mechanism, but rather as a complex regulation of the different connected modules inside the network.

### Regulatory Bioleaching Modules

As shown in Figure 3, there were eight transcription factors (dark-gray boxes) that were not controlled by any other regulator inside the co-regulatory network. We referred to them as *primary regulators* in our model, representing the first modular structure inside the network. For each primary regulator, its potential to regulate metabolic operons of each bioleaching-related pathway, either by direct binding or by chain regulatory cascades, was computed (Table 1). We found no clear exclusive distribution between primary regulators and metabolic bioleaching sub-categories. Most of these transcription factors regulate at least one operon of each sub-category, and, with the exception of primary regulators CysB and CynR and the energy generation sub-category, most primary regulators could not regulate an entire sub-category. These results reinforce the idea that regulation of bioleaching metabolism has a complex modular structure, where an important part of operons related to bioleaching processes are transcriptionally controlled by different primary regulators.

**Table 1:** Transcriptional regulatory representation of each primary regulator over the metabolic bioleaching sub-category.

	**Operons**	**CysB**	**SMTB**	**CynR**	**ArsR**	**CusR**	**Fur**	**Fis**	**IscR**
NAD biosynthesis	3	1	3	1	1	0	0	0	0
Heme biosynthesis	5	5	3	3	3	2	2	1	1
Spermidine biosynthesis	7	5	3	2	3	2	1	2	1
Sulfur assimilation	11	9	8	7	7	4	4	5	5
Energy generation	13	13	10	10	9	9	9	7	5
RISC oxidation	22	17	13	13	12	12	10	11	8
TOTAL	61	50	40	36	35	29	26	26	20

*Column Operons corresponds to the total number of operons for each metabolic bioleaching sub-category. The rest of the columns show the number of operons regulated by each listed primary regulator (transcription factor)*.

In order to examine this point in depth, we analyze whether single primary regulators have some specific property to control an exclusive sub-category. Thus, for each sub-category, we computed the number of operons that were controlled exclusively by only one primary regulator (Table 2). The percentage of operons exclusively regulated by one primary regulator was slightly greater for the biosynthesis sub-categories (NAD, heme, and spermidine). In contrast, sulfur assimilation and RISC oxidation processes were mostly composed by operons regulated by two or more primary regulators. This observation suggests that metabolic processes related to bioleaching in Licanantay present a bias in the transcriptional regulation according to specific sub-categories. It was possible to identify at least two regulatory modules. The first one was composed of specific primary regulators controlling an important number of operons related to biosynthesis of molecules involved mainly in redox reactions (NAD) (Gazzaniga et al., 2009) and iron homeostasis (heme and spermidine) (Bergeron et al., 2001; Quatrini et al., 2005; Richard et al., 2019). Considering the elevated capacity to tolerate high amounts of metals and oxidative stress damage by Licanantay (Latorre et al., 2016), the presence of unique primary regulators controlling gene expression of resistance mechanisms, provides the system with a fast and efficient transcriptional activation. The second module was comprised of several primary regulators controlling the expression of different energy related processes. This configuration suggests an important regulatory redundancy inside this module. The involvement of different primary regulators grants the system alternatives to produce or consume energy, ensuring the correct functioning of Licanantay. This contrasts with the previous module of biosynthesis of resistance-related molecules, which was highly specific in terms of transcriptional and metabolic response.

**Table 2:** Total number of operons for each metabolic bioleaching sub-category regulated by only one primary regulator.

	**Operons**	**Total**	**CysB**	**SMTB**	**CynR**	**ArsR**	**CusR**	**Fur**	**Fis**	**IscR**
NAD biosynthesis	3	2 (67%)	0	2	0	0	0	0	0	0
Heme biosynthesis	5	2 (40%)	2	0	0	0	0	0	0	0
Spermidine biosynthesis	7	3 (43%)	2	0	0	0	1	0	0	0
Sulfur assimilation	11	3 (27%)	1	1	0	0	0	0	0	1
Energy generation	13	1 (8%)	1	0	0	0	0	0	0	0
RISC oxidation	22	7 (32%)	4	1	0	0	1	0	1	0

*Total percentage was calculated as the proportion between the operon regulated by only one primary regulator and the total number of operons belonging to each sub-category*.

In most cases, primary regulators are not able to control an entire bioleaching sub-category. Thus, an analysis of the regulatory capacity of small subsets of regulators was performed in order to determine the degree of regulation specificity of each sub-category. This was done by computing sets of minimum transcription factors able to control an entire sub-category (Supplementary Table 1). Results showed that, except for spermidine biosynthesis, there was a unique minimum set of primary regulators for each sub-category. This analysis also highlighted the transcriptional factor CysB as the most represented regulator connecting genes involved in the selected bioleaching metabolic pathways, appearing in almost all the six sub-categories inside the group of minimal regulators.

In addition to the analysis of primary regulators inside the network, it was also possible to classify transcription factors according to number of connections. Three regulators stand-out due to their elevated number of connections: CysB with out-degree 14, LysR with in-degree 9, and IHF with a total degree of 16 (in-degree 6 and out-degree 10). These transcription factors had an affinity with operons in different metabolic bioleaching sub-categories. As mentioned, CysB directly controlled 4 of them, IHF 5 and LYSR 3. Thus, CysB, IHF, and LysR were considered complex regulator modules. In addition, *ihf* and *lysR* genes were controlled by other 6 and 9 transcription factors, respectively. On the other hand, IHF regulated three other regulators (being one of them LysR) while LysR could also regulated PuuR. These two regulators have been widely studied in other bacteria species, both controlling central metabolism and general processes in the cell (Schell, 1993; Lynch et al., 2003). While IHF and LysR had a high connectivity in the network, neither were central to maintaining the connectivity of the network (not belonging to minimal sets of regulators). On the contrary, the transcriptional regulator CysB in all the topological studies made, and under all the different analyses was a fundamental module in the transcriptional regulation of Licanantay. As showed in Figure 3, CysB also belonged to the first hierarchical level of organization inside the co-regulatory network, positioning this regulator as the main module involved in bioleaching processes.

## Conclusion

The availability of genome sequences has opened an interesting field in systems biology to study global gene regulatory organization in bacteria of biotechnological interest. Through the identification of sets of confident regulatory relations, the integration of information from two biological network was achieved: (i) affinity transcriptional regulatory network and (ii) metabolic network. As a result, the first co-regulatory network model describing the global transcriptional regulation of different bioleaching metabolic pathways in the bacterium *A. thiooxidans* Licanantay was generated. The topological analysis of the network indicates that the global transcriptional regulation is a result of the combination of different specific modules. The first type corresponds to primary regulators (transcription factors not controlled by another regulator). Inside this group, CysB appeared as the most relevant module inside the network, also classified as the most represented primary regulator controlling a huge part of the network. Another two types of modules were identified in terms of bioleaching pathway regulation distribution. Metabolic processes involved in energy production demonstrated a complex integrated regulation, where different primary regulators controlled the expression of several genes (complex modules). In contrast, bioleaching pathways related to metal homeostasis and oxidative stress damage were mainly regulated by unique primary regulators (individual modules). The presence of both modules showed that at least two types of regulation were present in the bioleaching bacteria. Complex modules provide a wide set of alternatives related to energy requirements of the network. Individual modules on the other hand, highlight an efficient and specific metal resistance capacity to survive under the extreme environmental condition present in mines. These results bring us closer to having an complete view of *A. thiooxidans* metabolism and regulation. Moving forward and applying systems biology methodologies to the study of additional key bioleaching bacteria can inform and aid the rational design of effective biomining consortia for bioleaching processes. Finally, this integrative systems biology strategy should not be restricted to biomining related bacteria, but can also be applied to other sequenced bacterial genomes to construct new co-regulatory networks.

## Materials and Methods

### Genome Annotation and Metabolic Network Reconstruction

Coding sequences (CDSs) previously identified in *A. thiooxidans* Licanantay draft genome (Travisany et al., 2014) were re-annotated. This was done through Blast searches against the nr, KEGG, UNIPROT, and COG databases. A gbk file with this new annotation was generated and used as the input to generate an automatic metabolic network reconstruction using Pathway-Tools v 21.0 (Karp et al., 2002). The cutoff score used for pathway prediction was 0.4. Additionally, a set of metabolic pathways previously suggested as relevant in bioleaching processes (Latorre et al., 2016) as well as central metabolism pathways were manually curated (Supplementary Table 2).

### Affinity Transcriptional Regulatory Network

The generation of the Affinity transcriptional regulatory network was based on previously reported protocols (Latorre et al., 2014; DebRoy et al., 2016). Briefly, candidate transcription factors present in the genome of *A. thiooxidans* Licanantay were identified using the following protocol: First, using the results of genome annotation, each candidate must have at least a Helix-Turn-Helix domain, previously identified using HMMER software with Pfam database. Then, when information from UniProt-KB was available, amino acids in specific locations were manually searched (Supplementary Table 3). A position-specific scoring matrix (PSSM) was associated with each transcription factor candidate that fulfilled the previous requirements (Supplementary Table 4). Specifically, a Regprecise (Novichkov et al., 2013) or Prodoric (Münch et al., 2003) PSSM was downloaded or generated using MEME (Bailey et al., 2009) with promoter consensus sequences.

Operons and intergenic regions were retrieved from previous research (Travisany et al., 2014) in the following manner: an operon was defined as a cluster of co-regulated consecutive genes that share the same direction such that the maximum intergenic region between two consecutive genes contained <50 bp. Any region larger than 50 bp was considered a putative promoter intergenic region.

In order to identify putative binding sites for the transcription factor candidates, affinity relations between candidates and promoter intergenic sequences were obtained. To that end, individual occurrences of the associated PSSM motifs in the promoter intergenic sequences were computed using FIMO (Grant et al., 2011). Thus, an affinity relation between a transcription factor candidate and an operon was defined when at least one match (*p* ≤ 1e^−5^) of the PSSM associated with the transcription factor was obtained in the correspondent promoter region of the operon.

The affinity network is a directed graph that encompasses the set of all affinity relations. In this network there are two types of nodes: (a) transcription factor nodes that correspond to families of transcription factors and (b) operon nodes which are operons being regulated by transcription factors. Note that when there are several genes coding for transcription factors of the same family, there is only a single node representing the family in the affinity network. There are also two types of arcs: (a) arcs from a transcription factors node to an operon node, which indicate that there is an affinity relation between the transcription factors and the operon; and (b) arcs between two transcription factors nodes A and B, which indicate that there is an affinity relation between transcription factor A and an operon containing a gene which codes for a transcription factor B.

### Co-regulation Network

A set of transcriptional regulations was defined as a regulatory mechanism that coordinates the expression of operons related to bioleaching in *A. thiooxidans* Licanantay.

To obtain this set of regulations, metabolic operons from six pathways previously associated with bioleaching were selected (Latorre et al., 2016). These pathways are NAD Biosynthesis, Heme Biosynthesis, Spermidine Biosynthesis, Sulfur assimilation, Energy generation, and RISC oxidation. Operons from these pathways that are regulated by at least one transcription factor were identified in the affinity network.

Under the assumption that the co-expression of operons belonging to the same metabolic pathway must be coordinated by a common factor (by directly regulating their expression or by regulatory cascades of transcription factors) and using a methodology that maximizes parsimony, arcs that are likely part of this co-regulation were selected from the affinity network (Acuña et al., 2016). Following this methodology, arcs in the affinity network were classified in four groups of the same size according to the *p*-value computed for the corresponding binding (between the transcription factor and the operon target). Then, weights of 1, 2, 4, and 8 were associated with arcs in each one of the four categories (a weight of 1 was given to arcs with lower *p*-values and a weight of eight to arcs with higher *p*-values).

To find common regulators of bioleaching related operons, the concept of a V-shape was used, which has been previously defined (Acuña et al., 2016). A V-shape is a subgraph that connects two given nodes (A and B) in a graph. It is composed of the union of two directed paths ending, respectively at A and B and starting at some node C with no other node in common. If a V-shape exists that connects A and B, then its starting point is a common regulator candidate. If more than one V-shape exists, then a parsimonious solution should be a combination of selecting a V-shape that uses less arcs and a V-shape having arcs with the smallest *p*-values. A way to consider both criteria is to consider V-shapes of minimum total weight (considering the total weight of a V-shape as the sum of the weight of its arcs).

According to the method explained, the set of all minimum weight V-shapes connecting two metabolic operons of the same metabolic category was computed involving 498 affinity relations, all of them having an original *p* < 0.00010. A histogram of the *p*-value obtained for the 498 affinity relations showed a decreasing tendency in the interval [0–0.00008] and a high peak in the interval [0.00008–0.00010]. In order to assure a confident set of relations, we applied a correction by removing arcs with an original *p* >0.00009 from the network. As a result, a set of 457 arcs was selected from the affinity network, involving 63 operons coding for transcription factors, and 91 operons coding for metabolic genes. Operons coding for transcription factors were annotated according to the family of transcription factors they belong to, resulting in a total of 34 families. Finally, the co-regulatory network contained 95 nodes: 34 transcription factor family nodes and 61 metabolic operon nodes. Arcs in this network were defined according to selection by V-shapes. That is, if an arc from a transcription factor A to a target operon B was selected from the affinity network, then the co-regulatory network included an arc from the family of A to B (or to the family of B, if B itself was also a transcription factor). Thus, a total of 148 arcs were computed between the 95 nodes previously defined.

## Data Availability Statement

The raw data supporting the conclusions of this article will be made available by the authors, without undue reservation, to any qualified researcher.

## Author Contributions

MC, VA, and ML designed the research and analyzed the data. MC, DT, and VA generated the bacterial network models. AS and AM supported the bioinformatics metabolic and regulatory networks reconstruction, respectively. MC, DT, VA, and ML wrote the paper. AM and ML take responsibility for the manuscript. All authors read and approved final content.

### Conflict of Interest

The authors declare that the research was conducted in the absence of any commercial or financial relationships that could be construed as a potential conflict of interest.
